# Challenges in LC–MS-based metabolomics for Alzheimer’s disease early detection: targeted approaches versus untargeted approaches

**DOI:** 10.1007/s11306-021-01828-w

**Published:** 2021-08-28

**Authors:** Pierluigi Reveglia, Carmela Paolillo, Gabriella Ferretti, Armando De Carlo, Antonella Angiolillo, Rosarita Nasso, Mafalda Caputo, Carmela Matrone, Alfonso Di Costanzo, Gaetano Corso

**Affiliations:** 1 Department of Clinical and Experimental Medicine, University of Foggia, 71122 Foggia, Italy; 2 Policlinico Riuniti University Hospital, 71122 Foggia, Italy; 3 Department of Neuroscience, School of Medicine, University of Naples Federico II, 80131 Napoli, Italy; 4 Department of Medicine and Health Sciences, Center for Research and Training in Aging Medicine, University of Molise, 86100 Campobasso, Italy; 5 Department of Molecular Medicine and Medical Biotechnology, School of Medicine, University of Naples Federico II, 80131 Napoli, Italy

**Keywords:** Alzheimer’s disease, Biomarkers, Untargeted metabolomics, Targeted metabolomics

## Abstract

**Background:**

Alzheimer's disease (AD) is one of the most common causes of dementia in old people. Neuronal deficits such as loss of memory, language and problem-solving are severely compromised in affected patients. The molecular features of AD are Aβ deposits in plaques or in oligomeric structures and neurofibrillary tau tangles in brain. However, the challenge is that Aβ is only one piece of the puzzle, and recent findings continue to support the hypothesis that their presence is not sufficient to predict decline along the AD outcome. In this regard, metabolomic-based techniques are acquiring a growing interest for either the early diagnosis of diseases or the therapy monitoring. Mass spectrometry is one the most common analytical platforms used for detection, quantification, and characterization of metabolic biomarkers. In the past years, both targeted and untargeted strategies have been applied to identify possible interesting compounds.

**Aim of review:**

The overall goal of this review is to guide the reader through the most recent studies in which LC–MS-based metabolomics has been proposed as a powerful tool for the identification of new diagnostic biomarkers in AD. To this aim, herein studies spanning the period 2009–2020 have been reported. Advantages and disadvantages of targeted vs untargeted metabolomic approaches have been outlined and critically discussed.

## Introduction

Alzheimer's disease (AD) is a progressive untreatable neurodegenerative disorder, which impairs the integrity of brain cells, making gradually the individual who is affected, unable of a normal life. AD is the fifth leading cause of death for people aged 65 and over (Alzehimers Associacion, 2019). Presently, more than 47 million people are estimated to be living with dementia worldwide with a projection to rapidly reach 75 million by 2030 and 135 million by 2050 (Alzehimers Associacion, 2019). Characteristics of the disease are the progressive loss of memory, reasoning, judgment, and language, to such an extent that interferes with daily life and personal activities. Intermediate stages between normal ageing and AD, defined as subjective memory complaint (SMC) and mild cognitive impairment (MCI), have been studied and characterized (Rami et al., 2010).

**Fig. 1:**
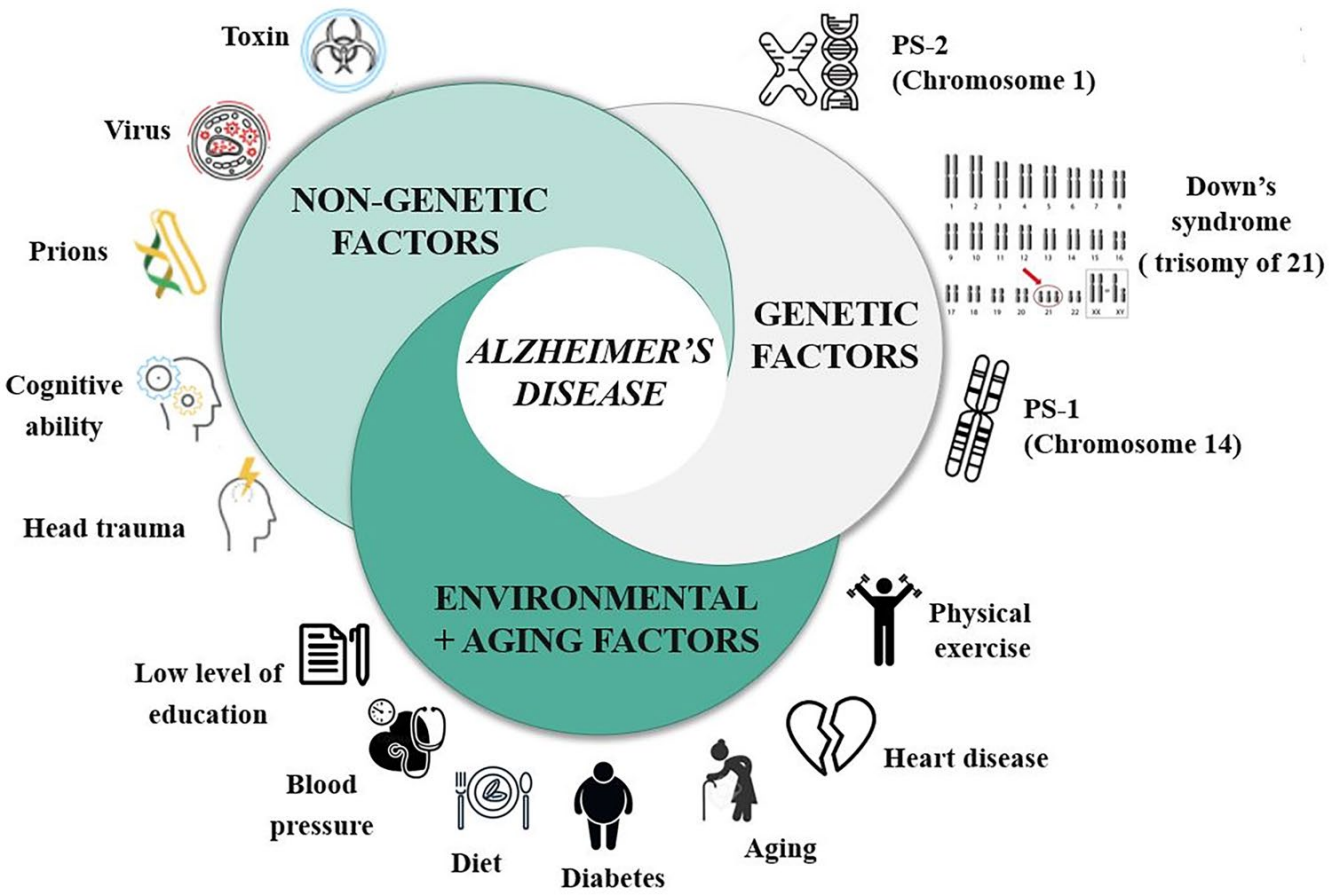
Risks factors in AD

AD is a complex disease and several could be the factors involved in its pathogenesis. These factors could be divided in three different groups: genetic factor, non-genetic factors and environmental and aging factors (Fig. 1).

The pathological hallmarks in AD are the formation and brain accumulation of extracellular beta-amyloid (Aβ) deposits, as oligomeric soluble structures or plaques, and intraneuronal neurofibrillary tangles containing hyperphosphorylated tau protein (Huang, 2012). Aβ aggregates and tau tangles promote synaptic deficits and enhance inflammatory processes and oxidative stress (Reiman et al., 2006). Although the exact cause of AD is still debated, the accumulation of Aβ in the brain provided a rational basis to hypothesize that it represents an initiating factor in AD pathogenesis, activating a cascade of events that progresses with tau-tangle formation and ends in neurodegeneration (Attems & Jellinger, 2014; Bailey et al., 2004; Farkas & Luiten, 2001; Love & Miners, 2016; Matrone et al., 2008, 2009; Oakley, 2006). Nevertheless, the lack of correlation between Aβ deposits/tau tangles accumulation in brain and neuronal degeneration, the amyloidogenic hypothesis is still object of an extensively compelling research (Iannuzzi et al., 2020; La Rosa et al., 2015; Leonenko et al., 2019; Matrone, 2013; Matrone et al., 2019, 2020; Poulsen et al., 2015, 2017).

In line with the amyloidogenic hypothesis, in the early-onset familial AD (EOAD) forms genetic mutations in APP or Presenilin1/2 (Fig. 1) (http://www.alzforum.org/mutations) genes cause an increase in Aβ42 production with a higher propensity for aggregation and/or with changes in the ratio of Aβ42/Aβ40 formation (http://www.alzforum.org/mutations). (Zhang et al., 2017).

On the other side, the majority of AD cases (90%–95%) are late-onset Alzheimer disease, (LOAD) which are “sporadic” and with no apparent familial recurrence. (Van Cauwenberghe et al., 2016). Several other mechanisms, different from Aβ accumulation, have been also reported to contribute to LOAD, such as hypertension, dyslipidemia, hypercholesterolemia, IGF/insulin resistance disorders, obesity and diabetes, thus suggesting to refer to LOAD as a metabolic disease (Leonenko et al., 2019; Van Cauwenberghe et al., 2016).

Gender difference also has a considerable impact on the onset of AD. Indeed, more than 60% of AD individuals are composed of post-menopausal women, and the presence of ApoE4 allele makes women more vulnerable in developing AD early (Rahman et al., 2016). Moreover, many studies linking nutrients intake and the risk of AD are rapidly increasing in the literature (Angeloni et al., 2020; Bracko et al., 2020; Gu et al., 2010; Shishtar et al., 2020; Veurink et al., 2020).

### Progress in diagnostic criteria: biomarkers in Alzheimer’s disease

The latest National Institute on Aging and Alzheimer’s Association (NIA-AA) 2018 guidelines, have underlined the importance to assess three different biomarkers before AD diagnosis. This has been classified as the A/T/N system, where “A” refers to amyloid β (Aβ), that can be assessed by either PET imaging of amyloid plaques or cerebrospinal fluid (CSF) of Aβ42 or the Aβ42 to Aβ40 ratio; “T” refers to tau pathology and can be measured by CSF phosphorylated tau or tau PET imaging of parenchymal neurofibrillary tangles; “N” refers to neurodegeneration and can be evaluated as elevated levels of CSF total tau, decreased glucose metabolism detected by FDG-PET imaging, and brain atrophy by using structural MRI (Jack et al., 2018). Additionally, recent reports indicate that the pathophysiological process of AD is detectable in CSF and the imaging markers for up to 20 years before dementia appears. (Jack et al., 2018; Sperling et al., 2014). In fact, the challenge in effectively treating AD, and yet perhaps the greatest promise, consists in the possibility to find biomarkers for the early detection, allowing the design of a personalized therapeutic approaches for each patient and the monitoring of the therapeutic response during the treatment. To achieve this goal a powerful bioanalytical pipeline should be used (Hampel et al. 2018).

Metabolomics strategies have the power to compare the metabolome in biological samples under normal conditions with altered states promoted by diseases, including AD. Common analytical techniques used for this purpose are nuclear magnetic resonance (NMR) and liquid chromatography—mass spectrometry (LC–MS). Indeed, very recently, NMR-based metabolomics analysis of biological fluids has provided models to discriminate among subjects with different stages of AD (Di Costanzo et al., 2020). Nevertheless, in the last decade, there has been enormous progress in LC–MS-based metabolomics, providing researchers with a variety of choices for chromatographic separation, ionization, and mass analyzers. These technologies have been applied in several clinical investigations for the identification of potential biomarkers for different pathologies (An et al., 2020; Cho et al., 2020; Huang et al., 2020; Pinto et al., 2020; Tang et al., 2020; Wilkins, et al. 2018; Yin & Xu, 2014).

In the present review we focused our attention on the most recent metabolomics studies (2009–2020) oriented to the discovery of AD’s biomarkers using LC–MS platforms. We classified the studies according to the approach used, targeted or untargeted, highlighting advantages and disadvantages of both approaches. Moreover, we evaluate the perspective for metabolomics in the complex and important pursuit of biomarkers for AD. Dissecting metabolic differences in the biofluids of AD patients may prospect the possibility to identify specific pathways within specific subgroups of patients, guiding the way to a personalized medicine.

## Metabolomics studies

Metabolomics studies applying untargeted approach have been important for the selection of potential biomarker for AD (Wilkins et al., 2018). The wide number of metabolites that have been studied, especially using LC–MS/MS platforms, could help in shed light on alteration in metabolic pathway in AD paving the way to possible novel diagnostic tools. Herein, in almost all of the works carried out with metabolomics, the data collected were analyzed using multivariate statistical modeling.

### Untargeted metabolomics studies

Several previous studies have demonstrated the presence of altered lipid profile in different stages of AD. The first attempt to identify biomarkers using metabolomics in serum of AD patience was carried out by Purandare et al. in 2009. To achieve their goal, the authors used both a gas chromatography time of flight mass spectrometry (GC-TOF–MS) and a LTQ Orbitrap to carried out the complete metabolic profiling of the serum samples (Purandare et al., 2009).

In the same year, Greenberg and colleagues investigate potential biomarkers for AD in human plasma.by UPLC-QTOF-MS was performed on a small set of samples (28 AD and 10 controls). Due to the high levels of inter- and intra-subject variability and the small number of samples they were unable to identified a statistically significant biomarker. However, they identified several plasma metabolites worthy of further research, suggesting that lipid metabolites (including bile acids) could be promising biomarkers (Greenberg et al., 2009).

Nine potential biomarkers were identified by UPLC-QqQ-MS on plasma samples from 20 AD patients and 20 healthy controls. Among the potential target there were lysophosphatidyl cholines (LPCs), tryptophan, dihydrosphingosine, phytosphingosine and hexadecasphinganine (Li et al., 2010). Further investigation on AD’s lipid profiling has be done by Han et al. (2011). They investigated over 800 specific lipid molecules in 26 AD patients and 26 normal controls by multi-dimensional mass spectrometry (shotgun). Consistently with the previous findings, eight sphingomyelin species (containing long aliphatic chains), were significantly lower in AD compared to aged-matched controls (Han et al., 2011).

Oresic et al. (2011) were able to characterized AD patients by diminished ether phospholipids, phosphatidylcholines, sphingomyelins and sterols by UPLC-TOF–MS on plasma samples. They also showed the predictive power of 2,4-dihydroxybutanoic acid for AD progression (p = 0.0048) (Oresic et al., 2011).

Non targeted metabolomic approach based on capillary electrophoresis–mass spectrometry (CE–TOF–MS) has been used to generate a predictive model for Alzheimer disease. Ibanez et al. (2012) studied the metabolic difference in cerebral-spinal fluid samples obtained from 85 AD patients with different cognitive status (73 samples to build the predictive model and 12 samples for its validation). Their predicted model is based on 14 metabolites and has a reported diagnostic accuracy of 83%. Moreover, choline, dimethylarginine, arginine, valine, proline, serine, histidine, creatine, carnitine, and suberylglycine were identified as possible disease progression biomarkers (Ibanez et al., 2012).

Inoue et al. (2013) proposed a brain metabolic profiling using UPLC coupled with time-of-flight mass spectrometry analysis. Significant differences in the levels of spermine and spermidine were identified comparing data from 10 AD with 10 controls (Inoue et al., 2013).

Whiley et al. (2014) investigated plasma lipids in AD, the initial metabolic screening involving both UPLC-QTOF-MS, and NMR spectroscopy. Plasma from 3 different groups was screened: individuals with AD, individuals with MCI, and age-matched controls (NC). In details, the authors used 10 NC, 13 AD, 12 MCI for the screening phase, while 49 NC, 42 AD and 50 MCI were involved in the validation step. The authors detected abnormal phosphatidylcholine levels (PCs) in plasma of AD individuals. In detail, they identified 3 PCs: PC16:0/20:5 (p < 0.001), PC16:0/22:6 (p < 0.05), and PC18:0/22:6 (p < 0.005), that were not previously linked to AD that could possibly interact with brain amyloid deposit and have a crucial role in AD. These 3 PCs are therefore believed to be a potential biomarkers and easy target for biochemical assay development (Whiley et al., 2014).

In the same year, González-Domínguez and co-authors carried out the first comprehensive characterization of serum phospholipids alterations in subjects with AD. A total of 19 NC samples and 17 Ad samples were included in the study. The phospholipids profiling was carried out by shotgun metabolomics on UPLC-QTOF-MS platform (González-Domínguez et al., 2014). Furthermore, they used ICP-MS to detect phosphorus-containing compounds. As results, significant disorders in lipids concentration were detected, encompassing alterations in phosphatidylcholines, phosphatidylethanolamines, plasmenylcholines, plasmenylethanolamines and lysophospholipids.The authors proposed a panel of these phospholipids according to their VIP score and taking into account only variables with VIP values higher than 1.5. The authors proposed a multifactorial origin for these alterations involving overactivation of phospholipases, increased anabolism of lysophospholipids, peroxisomal dysfunction and inequalities in the levels of saturated/unsaturated ratio of fatty acids (González-Domínguez et al., 2014).

Wang et al. (2014) investigated altered metabolites in plasma samples of AD patients applied a comprehensive analysis using both UPLC-QTOF-MS platform and a GC-TOF–MS. In this study, 57 AD patients, 58 MCI patients, and 57 normal controls were included. ROC analysis and logistic regression were used for the data analysis to reveal the most qualified biomarker. A panel of six metabolites: arachidonic acid, N,N-dimethylglycine, thymine, glutamine, glutamic acid, and cytidine with an AUC of 1.00 were selected to discriminate AD subjects. While, five metabolites: thymine, arachidonic acid, 2-aminoadipic acid, N,N-dimethylglycine, and 5,8-tetradecadienoic acid with AUC of 0.998 (95% CI 0.993, 1.000) discriminate the MCI subjects. Furthermore, the authors carried out statistical analysis to find possible biomarker that can distinguish between subject with ApoEε4( +) mutation carriers and non-carriers. No differences in the plasma metabolic profiles in the AD group (p = 0.390) and in the MCI (p = 0.539), group compared to control subjects, was found (Wang et al., 2014).

Takayama et al*.* in 2015 (2015) described a new untargeted UPLC-QqQ-MS method for analyzing chiral metabolites, in particular aminoacids, in brain of 10 AD subjectes and 10 NC. Natural amino acids usually belong to the L-series, nevertheless the presence of the D-enantiomers can be related to a specific pathology. Takayama’s method relies on chiral derivatization of homogenate from AD’s brain followed by triple quadrupole-mass spectrometry analysis. The study highlighted that 9 compounds belonging to the class of carboxylic acid and 15 belonging to amines class, could be suitable biomarker candidates in the AD brain. However, validation steps are needed for this study (Takayama et al., 2019).

In the same year, Ansoleaga et al. (2015), analyzed the alteration of purine metabolism in brain of 58 AD patients compared with 34 control. They applied real time PCR (RT-PCR) for functional genomic and mRNA expression levels. UPLC-QTOF-MS platform was used to analyze brain extract samples. Alterations of purine metabolism’s enzymes were detected by RT-PCR. The statistical analysis of the QTOF data identified altered levels of dGMP, glycine, xanthosine, inosine diphosphate, guanine, and deoxyguanosine. The alteration of purine metabolism may affect the export of triphosphates nucleoside to extracellular space (Ansoleaga et al., 2015).

Paglia and co-authors studied alteration in post-mortem frontal cortex from AD’s patients by lipidomics and metabolomics using an UPLC-QTOF-MS platform for an unbiased approach (Paglia et al., 2016a). For this study a high resolution mass spectrometer in data-independent mode (MSE) was used. Thirty-four altered metabolites belonging to six metabolic pathways were capable to distinguish AD from negative control. In details, the metabolic pathways were: (i) alanine, aspartate, and glutamate metabolism, (ii) arginine and proline metabolism, (iii) cysteine and methionine metabolism, (iv) glycine, serine, and threonine metabolism, (v) purine metabolism, and (vi) pantothenate and CoA biosynthesis (Paglia et al., 2016a).

In the same year another study on serum samples of 75 subjects with AD, 17 MCI and 45 NC was conducted by Gonzalez-Dominguez et al. (2016). To achieve this goal, the samples were investigated by UPLC-QTOF-MS. Compounds were annotated by matching the high-resolution mass data with those available in metabolomics databases. After statistical analysis the most relevant alterations in AD subjects several discriminant metabolites were found for the first time in the phospholipids and sphingolipids metabolism. In particular, decreased levels of oleamide (p = 0.025), histidine (p = 0.039) and monoglycerides together with increased level of phenylacetylglutamine were also found in AD subjects. The authors suggested that the lower levels of histidine are probably due to its involvement in the anti-inflammatory response to the disease (Gonzalez-Dominguez et al., 2016).

Liang and co-authors investigate the differences in saliva metabolites from MCI and age-matched AD subjects using an untargeted metabolomic approach based on fast ultra-high performance liquid chromatography coupled with TOF (FUPLC-TOF–MS) (Liang et al., 2016). For this study 583 saliva samples from subjects with MCI and 660 samples from AD patients were investigated. The statistical analysis disclose a total of 10 metabolites can distinguish among AD and the MCI group. These candidate biomarkers were highlighted using VIP-score (VIP > 12 and p < 0.01): Cytidine (VIP = 14.34), Sphinganine-1-phosphate (VIP = 40.45), 3-dehydrocarnitine(VIP = 21.18), Phenyllactic acid (VIP = 35.11), Pyroglutamic acid (VIP = 16.96), L-glutamic acid (VIP = 16.80), Ornithine (VIP = 36.62), L-tryptophan (VIP = 12.66), Inosine (VIP = 25.88) and Hypoxanthine (VIP = 18.29. In addition, the author performed a ROC analysis and Cytidine, sphinganine 1-phosphate, and 3-dehydrocarnitine had the areas under curve (AUC) values of 0.995, 0.934, and 0907, respectively, showing an high discrimination power and confirmed the potential value for diagnosing AD. In particular, cytidine had a sensitivity of 99.7% and a specificity of 97.3% for diagnosing AD (Liang et al., 2016).

Proitsi and colleagues, recently performed the largest untargeted plasma lipidomics investigation for AD biomarkers (Proitsi et al., 2017). They performed the analysis on a UPLC-QTOF-MS system and data analyzed by univariate and multivariate analysis methods. MRI of whole brain of AD patients was also performed. As result, cholesteryl esters/triglycerides and phosphatidylcholines were related to disease progression and brain atrophy. One of the metabolites strongly associated with AD was the PC 40:4. In addition, an unknown compound with m/z 367, was associated with an increased risk for AD. All together, these findings helped to extend knowledge of AD progression mechanisms (Proitsi et al., 2017).

An untargeted metabolomics on saliva samples was carried by Huan et al. (2018) using a HPLC-FTICR-MS platform. A total of e 109 samples were analyzed, in details 35 CN, 25 MCI, 22 AD in the screening phase and 10 CN, 10 MCI, 7 AD for the validation phase. Metabolites from NC, MCI and AD subjects were derivatized by dansylation. The metabolites where identified by the comparison of the spectra with those already reported in the human metabolome database (HMDB) and in the evidence-based metabolome library (EML) using the software My Compound ID. The data analysis was conducted using machine-learning statistical techniques. With this workflow the authors (Huan et al., 2018) highlighted clear differences of metabolic changes across the three clinical conditions. However, the separation between NC and MCI samples was not significant. Phenylalanyl-proline, urocanic acid, phenylalanyl-phenylalanine, and tryptophyl-tyrosine (95% CI 0.711–0.939) were capable to diversify the AD from the NC. While phenylalanyl-proline, alanyl-phenylalanine and phenylalanyl-glycine (95% CI 0.743–0.986) ratio differentiated AD from MCI. This study on salivary biomarker provided insight in AD development and was an excellent opportunity for clinical applications (Huan et al. (2018).

In 2019, lipidomics analysis of serum samples from the Alzheimer’s Disease Neuroimaging Initiative baseline (ADNI) cohort 1 (226 NC, 392 MCI and 188 AD) was carried out by Barupal and co-authors using a UPLC-QTOF-MS (Barupal et al., 2019). The study was performed to investigate metabolic disorders that might contribute to AD onset and development. In details, the authors investigated both individual lipid and sets of lipids to find out eventual correlations with disease diagnosis, CSF markers of disease αβ 1–42, CSF total tau and cognitive decline and brain atrophy. For the data analysis the authors applied Spearman-rank correlation–based matrices and Kolmogorov–Smirnov test for P-value distributions. The statistical analysis highlighted that free fatty acids and acylcarnitines were positively correlated with both SPARE-AD and total tau in CSF. For these metabolites, the average Spearman correlation coefficient *rho* across sets was 0.63 with a vary between 0.19 < ρ < 0.82. This finding fortify the idea to use these serum lipids as biomarkers for neurodegeneration. Moreover, from the same analysis, notable associations of omega-3 and omega-6 lipids levels and AD diagnosis was found. Finally, the authors suggested the roles of genetic variations, drugs, and diet on the metabolism of MUFA and PUFAs in AD should be further investigated (Barupal et al., 2019).

### Targeted metabolomics studies

Targeted assays are focused on a small panel of promising metabolites whose chemical identity is known before data acquisition, and their absolute quantitation is accomplished by isotopically-labelled internal standards or by using the standard addition method (Broadhurst et al., 2018). Even though this approach is not suitable for the discovery of novel biomarkers, it is appropriate for the validation of previously proposed.

Czech et al. (2012) performed a metabolic target analysis on 79 AD and 51 healthy controls by using HPLC-QqQ-MS. Their analysis showed that increased cortisol and cysteine level are related to AD, as well as reduced level of uridine. Specificity and sensitivity was increased when the 3 biomarker were used together to identify AD cases (Czech et al., 2012).

Liu et al. studied the metabolic profile of two groups of subjects (23) affected with AD with different mean age: 80 or 60 years. The authors used HPLC–MS/MS platform to asses L-arginine content in different brain tissues. The study highlighted that concentrations of L-arginine were altered in relationship with age and AD. In addition, the activity of nitric oxide synthase and arginase were altered with age and in AD in a region-specific manner of the brain. However, the authors suggested that more in depth studies are needed (Liu et al., 2014).

The previous study highlighted the possible importance of lipids alterations in the AD’s development. Thus, lipid metabolites appear to be highly useful to develop diagnostic tools for AD and MCI.

Klavins et al. (2015) applied targeted quality-controlled metabolomics approach using the absolute IDQ p180 Kit using a UPLC-QTrap-MS. The authors of the study analyzed a high number of plasma samples from (35) NC, (33) MCI and (43) AD subjects. Lipids concentrations were capable to differentiate controls from MCI and AD. Furthermore, the ratio of PC 34:4 and lyso PC 18:2 differentiates controls from MCI (p = 0.0000007; area under the curve (AUC) under ROC = 0.85) and from AD (p = 0.0000009; AUC under ROC = 0.82) significantly (Klavins et al., 2015).

To gain further knowledge on late-onset AD (LOAD) Wood et al. (2016) investigated the plasma levels of diacylglycerols and ethanolamine plasmalogens of a wide cohort of patients (51 NC, 77 MCI and 90 AD) using high resolution Orbitrap-MS. Interestingly, the lipidomics analysis was capable to clearly differentiate both MCI and LOAD subjects. The patients were clustered in three groups: (i) subjects with lower circulating ethanolamine plasmalogen levels; (ii) subjects with higher plasma diacylglycerol levels; and (iii) patients with neither of these lipid alterations. Nevertheless, the data need further validation as also suggested by authors, because in the pilot study of a small patient cohort they only detected an increase of DAG levels in MCI patients but failed to detect any patients with plasmalogen deficits. (Wood et al., 2016).

In 2014, Mapstone and co-authors proposed 10 plasma phospholipids, as a possible biomarker for memory impairment in older adults (Mapstone et al., 2014). Casanova et al (2016) wanted to validate the Mapstone’s results using two large independent longitudinal studies of AD’s patients’ groups. They tested the metabolites by both flow injection analysis mass spectrometry (FIA-MS/MS) and HPLC-QTrap-MS. In addition, they also performed the analysis of other 187 metabolites without a priori hypotheses. Acylcarnitines, hexoses, amino acids and biogenic amines were analyzed by quadrupole-ion trap mass spectrometer. The authors were unable to replicate the Mapstone’s results in the samples. However, from the unbiased analysis of the other metabolites the phospholipids with fatty acid chains from C30 to C44 carbon–carbon bonds, appeared to be important for the alterations in distinct metabolic pathways (Casanova et al., 2016).

Metallomics can be defined as the extensive analysis of metal and metalloids species present in a biological matrix. In this specific context, Paglia et al. (2016b) conducted a study aimed to develop an approach to detect the variation of serum elements in neurodegenerative processes using an ICP-MS. Four different groups were studied: 24 patients with subjective memory complaint (SMC), 20 subjects with MCI, 34 subjects with AD and 40 healthy subjects. The serum analysis was conducted by ICP-MS. A cohort of six essential elements (manganese, iron, copper, zinc, selenium and calcium), toxic elements (such as mercury, vanadium, uranium, arsenic, strontium and tin) and their ratios were analyzed by a multivariate statistical model. In details, manganese, iron, copper, zinc, selenium, thallium, antimony, mercury, vanadium and molybdenum changed significantly among the four groups. Most of essential elements increase in SMC, while progressively decrease in MCI and AD. Toxic elements show a variable behaviour, since some elements tended to increase, while others tended to diminish in AD. Both essential, such as Se, Zn, and Mn, and toxic elements, such as V, Sr, Sn and U, strongly influenced the grouping of AD samples. Regarding the other groups Cu is a potential candidate to discriminate SMC from HS, while Mn, Se and Zn appear to be able to discern between MCI and SMC (Paglia et al., 2016b). For validation the authors selected the biomarkers with the (AUC) higher then 0.7 and statistical power higher than 70%. As general trend, AUC and p values resulted higher for ratios. Mn (AUC = 0.89) and V (AUC = 0.83) had the highest diagnostic power in the distinction between AD and HS. While Mn (AUC = 0.89) and the ratio Cu/Mn (AUC = 0.93), were ndividuated as appropriate biomarkers for discriminating NC from AD patients. These results supported the hypothesis that both toxic and essential metals could have an important role in the development of AD, although further validation steps are needed (Paglia et al., 2015, 2016a).

Cristofano et al. (2016) investigated the amount of free L-carnitine, acetyl-L-carnitine and acyl-L-carnitines in the serum of 24 SMC, 18 subjects with MCI, 29 subjects with AD and 46 healthy subjects. The analysis of the sera were carried out on QTrap-MS instrument using labelled internal stadard for acyl-L-carnitines. The statistical analysis highlighted no metabolites changed significantly between SMC and MCI. Twelve metabolites and 3 molar ratios were identified by PLS-DA as VIP with a score > 1.3, showing significant alteration in the content of different L-carnitine, acetyl-L-carnitine and acyl-L-carnitine in Alzheimer’s disease groups. In details, serum acetyl-L-carnitine and acyl-L-carnitine (C3-DC, C5-OH, C6:1, C10, C12, C12:1, C14, C14:1, C16:1, C18, C18:1 and C18:2) decreasing from HS through SMC and MCI up to AD patients. ROC curves showed that the diagnostic accuracy of acetyl-L-carnitine was very good (AUC = 0.82) and that of other acyl-carnitines, such as C12, C18:1 and C18:2, was good (AUC between 0.7 and 0.8), indicating that these metabolites could be proposed as potential biomarkers for the diagnosis of AD. These lower levels of acyl-L-carnitines might be correlated with pertubation of transport of fatty acids into the mitochondria resulting in impaired energy metabolism. However the authors suggested that further validation studies are needed before clinical applications (Cristofano et al., 2016).

Corso et al. (2017) studied the alterations of serum amino acids contents in patients over the course of development of AD diseases, with the main goal identify possible diagnostic biomarkers. 24 subjetcs with SMC, 18 subjects with MCI, 29 subjects with AD and 46 healthy subjects were involved in the study. The analysis of serum samples was conducted by FIA-QTrap-MS. For the data analysis the authors used a ROC curve-based model evaluation (Tester). The model showed that the 10 biomarkers had a very good diagnostic power with an AUC of 0.958 in discriminating AD from HS. In details, the multivariate model comprise 6 amino acids (Glu, Asp, Phe, ASA, HomoCit, and Cit) and 4 ratios (Glu/Cit, Cit/Phe, Xle/Phe, and Arg/Phe) that were capable of discriminated AD patients from healthy subjects with about 96% accuracy. Furthermore, the study highlighted that the content of citrulline, argininosuccinate, and homocitrulline increase with progression of the disease. The detection of amino acids concentration may assist the characterization of patients metabotype during the progression of AD and monitoring their variation may help to detect at-risk individuals (Corso et al., 2017).

Chouraki et al. (2017) proposed four biomarkers candidates for AD diagnosis. A total of A total of 2067 participants were followed over an average period of 15.8 ± 5.2 years, at the end of the monitoring period only 68 subjects developed AD. The data were collected by an UPLC-QTrap-MS. The plasma levels of 217 metabolites were measured in the participants to evaluate the dementia risk. Plasma anthranilic acid and homocysteine levels (95% CI 1.15–1.70; p value = 8.08 × 10 − 4) were significant associated with risk of incident dementia: the risk increased by 40% for an increase of one standard deviation. Furthermore, applying a more liberal p value threshold of 10–2, other three additional metabolites were individuated: glutamic acid (95% CI 1.11–1.72; p value = 3.80 × 10^−3^), taurine (95% CI 0.60–0.92; p value = 6.91 × 10^−3^), and hypoxanthine (95% CI 0.60–0.92; p value = 6.93 × 10^−3^). The authors also highlighted the potential neuroprotective role of uric acid and taurine (Chouraki et al., 2017).

Considering that drugs may affect metabolism, John-Williams et al. (2017) developed a dataset starting from serum samples from ADNI 1 (199 control, 356 MCI and 175 AD subjects) cohort where they include information on medications taken by the patients. The dataset was developed to assist pharmacometabolomic investigations and the discovery of metabolic failures correlated with AD. The analysis was carried out by UPLC-QqQ-MS platform. The data set can be accessed through Sage Bionetworks’ Synapse platform (Jhon-Williams et al., 2017). Indeed, medicinal can results in variation of metabolites levels, thus the authors generate a statistical method, in R environment, for automated curation of metabolomics data. The scripts allow the removing of samples that have missing clinical data or the deletion of analytes with poor precision. However, the analysis suffered of some limitation as also stated by the authors. For instance, the low mass resolution of QqQ-MS used for collecting the data was unable to resolve the peak on isomeric or isobaric level. Another weakness is that the curation workflow is very stringent, and thus good measurements can be excluded from the analysis, precluding correlational studies.

Marksteiner et al. (2018) used UPLC-QTrap-MS platform to quantify 20 bile acids. The metabolites were quantitatively analyzed in plasma of 30 healthy subjects, 20 MCI and 30 AD patients. The levels of lithocholic acid were significantly higher (50 ± 6 nM, p = 0.004) in plasma of AD patients compared to healthy controls (32 ± 3 nM). Levels of glycochenodeoxycholic acid, glycodeoxycholic acid and glycolithocholic acid were significantly enhanced (p < 0.05) in AD patients compared to MCI. All other cholic acid studied were not significantly different among the three class of subjects. The authors also conduct ROC analysis, the results showing that lithocholic acid showed an AUC of 0.689 (95% CI 0.556–0.822). However, the study showed low diagnostic accuracy and could be considered a pilot study (Marksteiner et al., 2018).

A very interesting parallel metabolomics analyses was recently carried out by Varma and colleagues using both FIA- MS/MS and HPLC-QTrap-MS (Varma et al., 2018). The study was divided into two phases and accomplished in both brain and blood samples to identify systemic changes of metabolites quantity during AD progression. For phase 1, 44 samples (14 CN, 15 asymptomatic Alzheimer’s disease (ASYMAD) and 15 AD) were collected from Baltimore Longitudinal Study of Aging (BLSA), for the validation phase both BLSA (115 NC and 92 AD) and ADNI (216 NC, 366 MCI and 185 AD) were used. After data collection, 26 metabolites capable to discriminate between AD and HC subjects were selected using machine-learning methods. The main results of the study showed that sphingomyelins (SM), hydroxy-sphingomyelins and glycerophospholipids (PC) were closely associated with the extent of AD pathology and progression. Moreover, the authors identified specific SM and PC through machine-learning methods to generate an AD-specific brain metabolite signature, and then clustered them to map the key biological pathways implicated in AD pathogenesis including tau phosphorylation, Aβ metabolism, calcium homeostasis, acetylcholine biosynthesis, and apoptosis. The study should be validated using a wider cohort of subjects (Varma et al., 2018).

In the same year Muguruma and colleagues proposed targeted metabolomics method for the evaluation of 97 amines in post-mortem CSF (pCSF) (Muguruma et al., 2018). 10 healthy subjects and 10 AD patients were included in the study. The authors performed the analysis on UPLC-QqQ-MS. The study identified several alterations in the concentration of metabolites belonged to polyamine and tryptophan-kynurenine (Trp-Kyn) pathways in patients with AD. In details, The ROC curves analysis of showed AUC values of 0.91, 0.90, 0.81, and 0.81 for Tryptophan, anthranilic acid, kynurenine and 3-hydroxykyurenine, respectively. In addition, abnormal levels of methionine sulfoxide, 3-methoxy-anthranilate, cadaverine and guanine in the pCSF of AD subjects were found (Muguruma et al., 2018).

Nho et al. (2019) investigated the possible connection between peripheral metabolic concentration and central biomarkers for AD pathophysiology (Nho et al., 2019). For the analysis samples from ADNI cohort were selected: 370 control, 98 SMC, 789 MCI and 305 AD. Compounds belonging to primary and secondary bile acid (BA) were evaluated by LC–MS/MS in serum samples. Other parameters were also evaluated during the study such as brain atrophy (magnetic resonance imaging) and brain glucose metabolism. The main results of the study showed that abnormal bile acid (BA) profiles were remarkably associated with structural and functional changes in the brain as recognized by larger atrophy and reduced glucose metabolism. Moreover, three BA ratios were highly associated with three CSF biomarkers including lower CSF Aβ1-42 levels (amyloid-β positivity) in addition to reduced cortical glucose metabolism and larger structural atrophy: Glycodeoxycholic acid/Cholic acid, Taurodeoxycholic acid/Cholic acid, and Glycolithocholic acid/Chenodeoxycholic acid. In conclusion, the authors suggested that BA signalling pathways could provide useful insights for the identification of protective metabolites against AD. However, further validation steps are required (Nho et al., 2019).

Mahmoudian Dehkordi et al. (2019) studied the stored blood samples from ADNI cohort (370 control, 789 MCI and 305 AD) to investigate the possible correlation of microbial imbalance and AD pathogenesis. In addition, the high number of studied samples allow to investigate the eventual correlation between this imbalance and innate immunity–related genes. Moreover, the authors also checked for medication use which is known to remarkably affect the gut microbiome and bile acids. Using a UPLC-QqQ-MS the authors found that the BAs profile was appreciably altered in AD subjects. In details, they detected a significant decrease in levels of the primary BA, Cholic acid (0.85, 95% CI 0.78–0.92; p 1.56E-04) produced by liver, while a significant increase of bacterially produced secondary BA, deoxycholic acid was noted (1.24, 95% CI 1.11–1.39; p 1.61E-04) along with several secondary conjugated BAs: glycodeoxycholic acid (1.30, 95% CI 1.17–1.43; p 4.20E-07), taurodeoxycholic acid (1.19, 95% CI 1.08–1.30; 3.26E-04), and glycolithocholic acid (1.33, 95% CI 1.20–1.48; p 9.21E-08). Furthermore, the authors also correlate the serum BAs concentration with CSF and neuroimaging biomarkers for AD. As results, they found a possible metabolic link between immune system and gut microbiome dysregulation and the increased production of cytotoxic secondary bile acids in AD subjects. In this frame the BAs represent a constituent of the gut-liver-brain axis that relates to cognition. Lastly, the authors highlighted the need of longitudinal studies covering pre-symptomatic stages to denote the influence of immune changes on gut microbiome composition and activity in AD subjects (MahmoudianDehkordi et al., 2019).

More recently, Huynh et al. (2020) carried out one of the most comprehensive lipidomic study of AD to date. They used HPLC-QqQ-MS platform to quantify 569 between lipid and lipid-like compounds from 32 classes and subclasses, underling the importance of analyze the whole lipidome at molecular structural detail to identify crucial lipid pathways involved in AD and its future onset. The authors applied their workflow to two large independent studies: The Australian Imaging, Biomarkers and Lifestyle (AIBL: 696 NC, 268 AD) and ADNI cohort (210 NC, 178 AD). After covariates (including age, sex, body mass index, total cholesterol, HDL-C, triglycerides, site of sample collection, APOE ε4 alleles, omega-3 supplementation and statin use) corrections, using a multivariate modelling to identify lipids important for AD diagnosis or predicting of future AD onset the authors observed a final concordance statistic (C-statistic) of 0.752 (95% CI 0.747–0.757) through the incorporation of 10 lipid species in the model. While, in the parallel analysis, where the ADNI was the discovery and AIBL was the validation, the disease classification model had a final C-statistic of 0.869 (95% CI 0.866–0.871). The predominately altered classes were the following sphingolipids: dihydroceramides (dhCer), trihexosylceramides (Hex3Cer), GM3 gangliosides (GM3), GM1 gangliosides (GM1). This was also the first report of an association between circulating GM3 gangliosides and AD. Moreover, also other lipids classes were altered: alkylphosphatidylcholine [PC(O)], alkenylphosphatidylcholine [PC(P)], alkylphosphatidylethanolamine [PE(O)], alkenylphosphatidylethanolamine [PE(P)], alkyldiacylglycerol [TG(O)]. The authors also performed further adjustments for MCI subjects, and in this case only the dehydrocholesteryl ester (DE 18:1) and two plasmalogen species had a significant association. Even though, this study improved the knowledge in the lipidomic area of research validating the results on two large cohorts, a population study will be required to fully assess model performance (Huynh et al., 2020).

## Comparison between targeted and untargeted approaches

The studies reviewed in the previous sections describe the most recent advances in the use of metabolomics to study diagnostic biomarkers in biofluids and tissues from patients with AD. It is important to highlight that particular care must be taken when human post-mortem brain tissue where selected for investigations. Indeed, not all post-mortem tissues are satisfactory for DNA, miRNA and protein studies. Moreover, metabolic pathways alterations are caused by several factors, but mostly by proteins expression levels (Ferrer et al., 2008). Thus, these samples must be cautious selected prior to use, and a possible pre-analytical bias, such as storage time, should be considered and assessed.

Moreover, the metabolome is composed by many classes of compounds with diverse chemical properties. For these reasons, global extraction of all metabolites in a given system is challenging, and the analyses, applying only one platform, is almost impossible. The metabolome is often roughly partitioned by polarity during the extraction process, and, in this context the “lipidome” and “metallome” can be considered as fractions of the whole metabolome.

The term “Lipidomic” was introduced for the first time to describe the whole complete set of lipid species existing in a cell, an organ, or a biological system (Han & Gross, 2003). Studying the lipidome is fundamental because it varies with time and with the different perturbations experienced by the organism. At present, lipidomics has become one of the most important branches of omics, is a very active research field. From the analytical point of view the most common platform used are LC–MS-based techniques (Wang et al., 2019). Nevertheless, due to the wide diversity of the lipid structure, there is still room for upgrade and refine the lipidomic approaches from sample preparation to MS analysis and data processing and analysis (Wang et al., 2019).

The term, “metallomics” was a newly proposed word to describe a field of –omic sciences that provides a comprehensive analysis of metal and metalloids species present in a biological system (Shi & Chance, 2008). Metallomics field includes various independent areas in trace metal investigation encompassing genomics, proteomics, and other omics-sciences. Mass spectrometry played an important role in metallomics. New generation ICP-sector field mass spectrometer for label-free detection of trace elements, HPLC-ICP-MS system with simultaneous/multielement detection could help to gain new insight in the science of biological trace metal (Haraguchi, 2017).

**Fig. 2:**
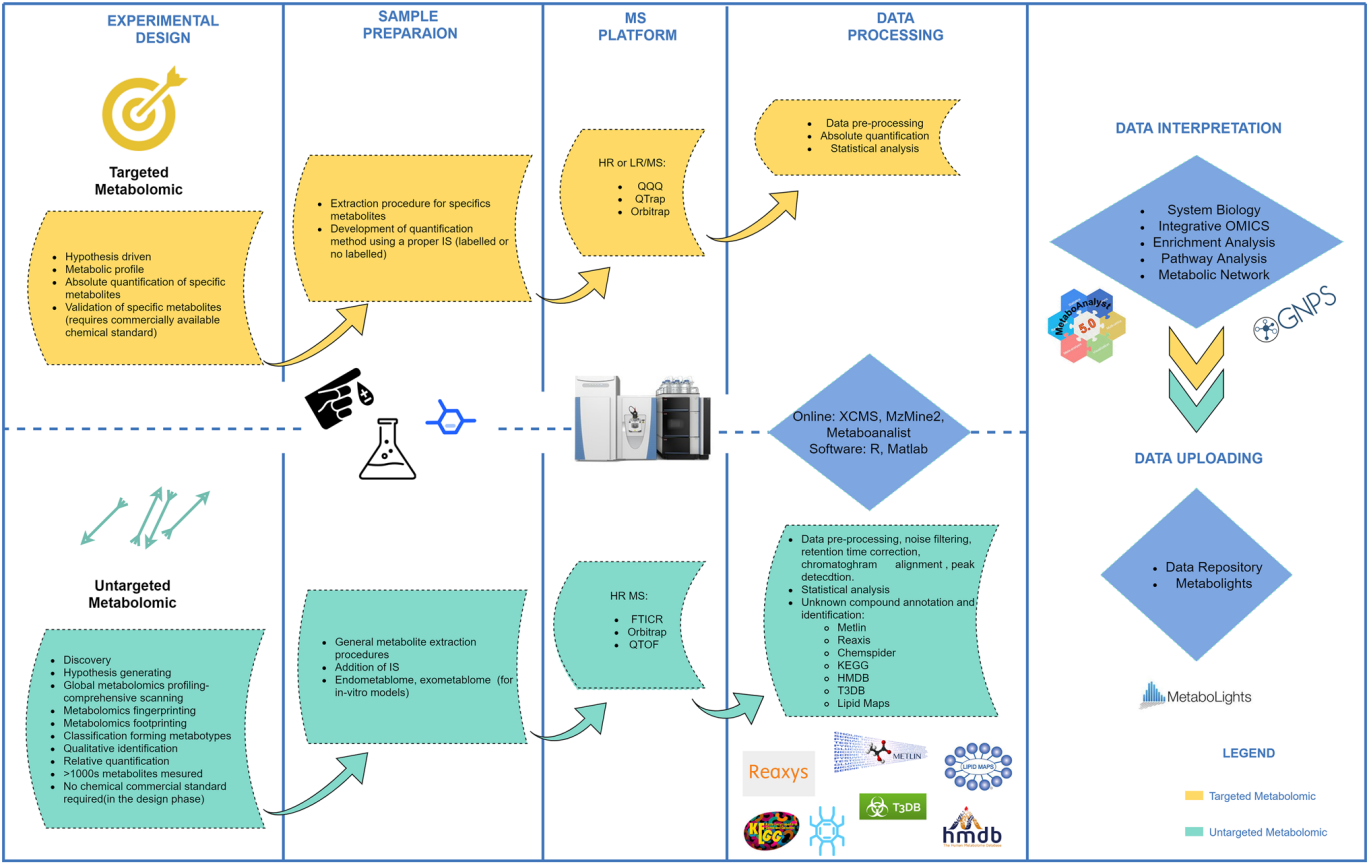
Untargeted and targeted metabolomics workflows using LC–MS platform

Although, there is still not an actual consensus regarding terminology used to classify metabolomics investigation, a straightforward and widespread definition that relates to the fact whether the researcher had or not a priori knowledge of the kind of metabolites to search (Broadhurst et al., 2018; Sussulini, 2017). Given the background, a targeted metabolomics approach is defined as a quantitative analysis (absolute concentrations) of a selected number of metabolites that might be correlated to common chemical classes or related to selected metabolic pathways or reaction (Broadhurst et al., 2018; Klassen et al., 2017). This approach is not suited for the discovery of novel compounds or metabolites involved in the studied process or diseases. In addition, to carry out the absolute quantitation proper internal standard or a labelled standard is required. This process is usually time consuming and expensive (Fig. 2).

Alternatively, the untargeted metabolomics assay could assist for the discovery of novel biomarkers through the reproducibly detection of as many metabolites as possible in a biological system. An untargeted metabolomics method relies on the qualitative or semi quantitative analysis of the largest possible number of metabolites from a diversity of chemical and biological classes present in a biological matrix. Both fingerprinting and footprinting metabolomics belong to this definition (Broadhurst et al., 2018; Klassen et al., 2017). The approach is unbiased and thus particularly suitable in AD, where relatively poor knowledge of pathophysiological processes occurring in the diseased brain. Unfortunately, the untargeted approaches did not lead to any validated biomarkers so far (Fig. 2). It is important to highlight that an intermediate assay between untargeted and targeted approaches is also commonly used and named semi-targeted assay (Broadhurst et al., 2018) In this approach, a priori known hundreds metabolites are targeted and relative quantifications (one calibrations curves and one internal standard for multiple metabolites) are accomplished.

For AD biomarker research, the predominant choice of MS, compared to NMR, as analytical platform is probably due to its higher sensibility, higher range of detectable metabolites and high throughput (Emwas et al., 2013). MS platforms applied for metabolomics investigation include low resolution (LRMS) such as QqQ-MS (Dawson et al., 2013) and QTrap-MS (Fortin et al., 2009; Li et el., 2019), or high resolution (HRMS) instruments, such as QTOF-MS (Marshall et al., 2008), also hyphenated with Ion mobility (IM) (Lapthorn et al., 2013), Q-Orbitrap-MS (Eliuk et al., 2015) and FTICR-MS (Hecht et al., 2006). The high resolution mass spectrometry is preferable when untargeted studies will be carried out. In fact, complex mixtures usually contain hundreds of metabolites with close mass differences and a resolution of at least 0.1 mDa is necessary to allow the separation of all the generated ions (Marshall et al., 2008). Indeed, sub-ppm mass accuracy is essential for the confident molecule annotation. Moreover, QTOF-MS platforms have been hyphenated with IM. The use of IM empowers ions separation according to their size and shape, allowing to differentiate isomeric and isobaric analytes. This assists a more robust day-to-day operation. Moreover, the separations in IM take place post-ionization and it happens in milliseconds, rather than seconds as in chromatography (Lapthorn et al., 2013). More recently, several ion mobility-mass spectrometry imaging experiments have been reported (Mesa Sanchez et al., 2020). Conversely, QqQ-MS or QTrap-MS are the work horse in targeted approach. Both instruments rely on MRM (Multi Reaction Monitoring) as their most sensitive and reliable method in the quantitation of metabolites. Nevertheless, HRMS is also capable of targeted metabolite quantification, QTOF-MS in MS1 mode was applied in different clinical studies that showed that its performance is comparable to traditional MRM assays on the QqQ-MS instrument (Ding et al., 2015; Gertsman et al., 2014). This assay strategy is usually called parallel reaction monitoring (PRM) (Lu et al., 2008; Zhou et al., 2016). PRM-based targeted metabolomics strategy was recently reported also for Q-Orbitrap equipment (Zhou et al., 2016).

Untargeted and Targeted metabolomics experiments differ in the flow of information and data processing (Fig. 2) (Goodacre et al., 2007). Prior to choose between the two metabolomics strategies, researchers need to focus on the scientific question they would like to address and a rigorous experimental design should be defined (Goodacre et al., 2007). Metabolomics analyses usually generate a large amount of data. To analyze this amount of data fast and accurate statistical and bioinformatics software or online platforms are used to generate biological information (Alonso et al., 2015). Matlab ad R sfotware, are widely used to carry out raw data processing and statistical analysis (Li et al., 2020). Among the available online platforms XCMS (Forsberg et al., 2018; Huan et al., 2017; Li et al., 2020) and Metaboanalyst are the two most widely used (Chong et al., 2019; Li et al., 2020).

For untargeted metabolomics studies features identification is also required, for such purpose, free databases and libraries, such as HMDB, KEGG, Reaxys, Chemspider, Metlin, or LipidMaps are used (Klassen et al., 2017). The listed metabolites are then used to statistically compare the analyzed samples and find significant impaired peaks for a given treatment allowing system biology investigation and multi-omics pathway analysis (Forsberg et al., 2018; Huan et al., 2017).

Although biological validation is not commonly pursued after completion of a metabolomics study, a validation step should be carried out to make a wider biological meaning of the results (Sussulini, 2017). Two different approaches could be used for validation: external validation in which an entire new set of samples are collected and processed; alternatively, internal validation could be carried out. The internal validation is a follow-up of the preliminary results of the untargeted metabolomics investigation, where selected metabolites are quantitatively analyzed in the same sample set. However, external validation is recommended (Sussulini, 2017).

Applications of current diagnostic tools strongly suggests that genes dysregulation, different expression of miRNA in AD subjects, and metabolic network alterations contribute to disease development (Navas-Carrillo et al., 2020; Wilkins et al., 2018). The altered patterns include post-translational functional modifications of proteins, lipid and amino acid and metals metabolism, and metabolic pathways involved in glucose and energy substrate utilization (Navas-Carrillo et al., 2020; Wilkins et al., 2018). Developing a multi-omics platform that links transcriptomic, proteomic, lipidomic, metallomics and metabolomics data can shed light on disease mechanisms. Analytical methods have been developed by omics scientists for multi-omics data acquisition. For instance, to merge proteomics and large-scale targeted metabolomics, Liu and coauthors proposed and verified liquid chromatography-hydrophilic interaction liquid chromatography-tailored selected reaction monitoring (RPLC-HILIC-tailored SRM) as a viable choice for large-scale targeted bi-omics (Liu et al., 2019). On the other hand, an equal number of efforts have been made by bioinformaticians to develop reliable workflows for system biology (Huan et al., 2017; Li, 2020). This joint endeavour may factually lead to novel biomarkers for the diagnosis, prognosis, and therapy monitoring.

Despite the impressive steps forward done in clinical metabolomics in the last decades, several pitfalls remain to be solved in the entire research workflow (Kohler et al., 2016). First, a proper experimental design is fundamental to obtain accurate data that could lead to meaningful biological results. Furthermore, optimal condition for collection, handling, storage, and preparation of the samples are also crucial for the analysis. Nevertheless, sample preparation is a challenging bottleneck because, usually, different steps are carried out by multiple research laboratories, and no standardized procedures are now available.

Unreliable sampling leads to high analytical variability increasing the systematic errors of data acquisition, hindering the comparisons between populations. The treatment of systematic errors is very challenging in untargeted MS-based metabolomics approaches where unknown metabolites are analyzed and potential data artefacts could arise. Data analysis became also more complex when multi-omics investigations are performed. Finally, great efforts are usually made for the discovery of novel biomarkers for the AD. The literature is overfull with biomarker candidates that have never reached the validation phase. Thus, multi-disciplinary studies and collaborations between institutions, pharmaceutical agencies, and companies, are crucial to validate and go beyond the actual limits.

Moreover, standardized guidelines for study design and standard protocols for sample collection, pretreatment and storage for multi-layer omics analysis should be established (Long et al., 2020).

## Perspective

Metabolites represent the final stage of genes- and protein-based processes. They can be studied through metabolomics workflow, disclosing the connection between the genotype and the phenotype (Fiehn, 2002). Metabolomics can monitor dynamic changes into biological systems suppling suitable information for clinical applications and translational medicine (Pagani et al., 2017; Trushina et al., 2013). The progress of “–omics” sciences gave a boost the development of the personalized medicine. This approach to medicine has the potential to transform healthcare, shifting from the present holistic focus to the individual focus. Indeed, “-omics” sciences are capable to take into account the individual differences in genetics, through genomics, and connect them to the phenotype, through metabolomics, lipidomics, and metallomics (Irvine & Nguyen, 2019). Nevertheless, future challenges for this science field will include harmonization of disparate datasets, protocols standardizations and subsequent algorithmic analysis to get novel insights. In fact, the Metabolomics Society with the Metabolomics Standards Initiative (MSI) and other authors supports the need for standardized reporting of metadata or information describing large-scale “-omics” data sets since 2007 (Sumner et al., 2007). In this regard, during the reviewing of LC–MS-based metabolomics studies presented in this manuscript we encountered little uniformity and relatively little rigor in how researchers select, assess or present their candidate biomarkers. This could give rise to complication in data comparison and validation. Thus, our hope is that all the researcher involved in the filed could adopt standard procedure to report and share their data following already proposed guidelines (Sumner et al., 2007; Xia et al., 2013).

Moreover, also the reliability and the reproducibility of extraction and analysis process still need to be improved and standardized. Indeed, the scientific community has made important advances in this regard (Long et al., 2020). It is important to highlight that the harmonization of laboratory procedures, such as the standardization of extraction protocol, sample’s matrices, and other pre- and post-analytical steps, is a fundamental step to obtain meaningful biological results (Emwas et al., 2013; Gelzo et al., 2012; Lindon et al., 1999; Long et al., 2020).

In AD research, several quantitative data have been collected on compounds suspected to play a role in the disease applying targeted approaches. Markers with different chemical nature including, biogenic amines, oxylipins, lipid mediators, amino acids, metals and oxidative stress markers have been identified as reported in Table 1. Two of the most recent works by Nho et al. (2019) and Mahmoudian Dehkordi et al. (2019) disclose the importance of the primary and secondary bile acids dysregulation in AD patients. Both studies used the large ADNI cohort for their investigation. These results are in agreement with the recent findings by Baloni et al. (2020) that studied alterations in cholesterol and bile acid metabolism in AD. The authors used a systems biology approach that reveal that taurine transport, bile acid synthesis, and cholesterol metabolism were abnormal in AD subjects. Furthermore, the concentration of some bile acids cannot be explained only by enzymatic synthesis, showing that they may be produced by the gut microbiome and then transported to the brain (Baloni et al., 2020).

**Table 1:** AD-related metabolomics studies for biomarker discovery from 2009 to 2020

Sample	Analytical platform	Number of samples	Proposed biomarkers	References
Untargeted metabolomics				
Plasma	UPLC-QTOF-MS	10 NC 28 AD	Lipid profile	Greenberg et al. (2009)
Plasma	UPLC-QqQ-MS	20 NC 20 AD	LPCs, sphingosine and tryptophan	Li et al. (2010)
Plasma	QqQ-MS	26 NC 26 AD	Sphingomyelins and ceramides	Han et al. (2011)
Plasma	UPLC-TOF–MS	46 NC 91 MCI 89 AD	Phosphatidylcholine, plasmalogens, sphingomyelins, sterols and Dihydroxybutanoic	Oresic et al. (2011)
CSF	CE-TOF–MS	Screening:73 AD (predictor model generation) Validation: 12 AD	Choline, dimethylarginine, arginine, valine, proline, serine, histidine, creatine, carnitine, and suberylglycine	Ibanez et al. (2012)
Brain tissue	UPLC-TOF–MS	10 AD vs. 10 controls	Spermine and spermidine	Inoue et al. (2013)
Plasma	UPLC-QTOF-MS	Screening:10 NC 12 MCI 13 AD Validation:49 NC 50 MCI 42 AD	Phosphatidylcholines (PC)	Whiley et al. (2014)
Serum	UPLC-QTOF-MS ICP-MS	17 NC 19 AD	Alteration in phosphatidylcholines, phosphatidylethanolamines, plasmenylcholines, plasmenylethanolamines	González-Domínguez et al. (2014)
Plasma	UPLC-QTOF-MS	57 NC 58 MCI 57 AD	Panel for AD: arachidonic acid, N,N-dimethylglycine, thymine, glutamine, glutamic acid, and cytidine Panel for MCI: thymine, arachidonic acid, 2-aminoadipic acid, N,N-dimethylglycine, and 5,8-tetradecadienoic acid for MCI	Wang et al. (2014)
Brain tissue; Cerebrospinal fluid	UPLC-QqQ-MS	10 NC 10 AD	9 Carboxylic acids 15 Amines	Takayama et al., (2015, 2019)
Brain tissue	UPLC-QTOF-MS	34 NC 58 AD	dGMP, glycine, xanthosine, inosine diphosphate, guanine, deoxyguanosine	Ansoleaga et al. (2015)
Frontal cortex	UPLC-QTOF-MS	19 NC 21 AD	Thirty-four altered metabolites belonging to six metabolic pathways	Paglia et al. (2016a)
Serum	UPLC-QTOF-MS	45 NC 17 MCI 75 AD	Oleamide, histidine, monoglycerides, phenylacetylglutamine	González-Domínguez et al. (2016)
Saliva	FUPLC-TOF–MS	583 MCI 660 AD	Cytidine, sphinganine 1-phosphate and 3-dehydrocarnitine	Liang et al. (2016)
Plasma	UPLC-QTOF-MS	152 NC 148 AD	PC 40:4	Proitsi et al. (2017)
Saliva	HPLC-FTICR-MS	Screening: 35 NC 25 MCI 22 AD Validation: 10 NC 10 MCI 7 AD	Phenylalanyl-proline, urocanic acid, phenylalanyl-phenylalanine, tryptophyl-tyrosine	Huan et al. (2018)
Serum and plasma	UPLC-QTOF-MS	226 NC 392 MCI 188 AD (ADNI Cohort)	MUFA-containing lipids were positively associated with the brain atrophy and tau accumulation. PUFA-containing lipids were negatively associated with AD	Barupal et al. (2019)
Targeted metabolomics				
Cerebral fluid	HPLC-QqQ-MS	79 AD vs. 51 controls	Combinations of three to five metabolites, including cortisol, cysteine, uridine and various amino acids	Czech et al. (2012)
Brain tissues	HPLC–MS/MS	23 NC 12 AD	L-arginine	Liu et al. (2014)
Plasma	UPLC-QTrap-MS	35 NC 33 MCI 43 AD	Ratio of PC 34:4 and lysoPC 18:2	Klavins et al. (2015)
Plasma	Orbitrap-MS	51 NC 77 MCI 90 AD	Diacylglycerol levels	Wood et al. (2016)
Plasma	HPLC-QTrap-MS	99 NC 93 AD (BLSA cohort)	Phospholipids with fatty acid chains from C30 to C44	Casanova et al. (2016)
Plasma	ICP-MS	40 NC 24 SMC 20 MCI 34 AD	Manganese, iron, copper, zinc, selenium, thallium, antimony, mercury, vanadium and molybdenum	Paglia et al. (2016b)
Serum	QTrap-MS	46 NC 24 SMC 18 MCI 29 AD	Acetyl-L-carnitine and acyl-L-carnitine levels	Cristofano et al. (2016)
Serum	FIA-QTrap-MS	46 NC 24 SMC 18 MCI 29 AD	Glutamate, aspartate, phenylalanine of citrulline, argininosuccinate, homocitrulline	Corso et al. (2017)
Plasma	UPLC-QTrap-MS	1974 NC 68 AD (FO cohort)	Anthranilic acid, glutamic acid, taurine, hypoxanthine	Chauraki et al. (2017)
Serum	UPLC-QqQ-MS	199 NC 356 MCI 175 AD (ADNI)	Generation of metabolomics dataset for applications in pharmacometabolomic investigation	John-Williams et al. (2017)
Plasma	UPLC-QTrap-MS	30 NC 20 MCI 30 AD	Glycochenodeoxycholic acid, glycodeoxycholic acid, glycolithocholic acid	Marksteiner et al. (2018)
Brain and blood samples	HPLC-QTrap-MS	Screening: 14 NC ASYMAD 15 15 AD (BLSA, Brain tissue); Validation:115 NC 92 AD (BLSA) 216 NC 366 MCI 185 AD (ADNI)	Sphingomyelin (SM) and hydroxy-sphingomyelin (H-SM)	Varma et al. (2018)
pCSF	UPLC-QqQ-MS	10 NC 10 AD	Methionine sulfoxide, 3-methoxy-anthranilate, cadaverine, guanine	Muguruma et al. (2018)
Serum	LC–MS/MS	370 NC 90 SMC 789 MCI 305 AD (ADNI)	Bile Acids ratios	Nho et al. (2019)
Serum	UPLC-QqQ-MS	370 NC 789 MCI 305 AD (ADNI)	Low concentrations of a primary cholic acid. Increased concentration of deoxycholic acid, and its glycine and taurine conjugated forms	Mahmoudian Dehkordi et al. (2019)
Plasma and serum	HPLC-QqQ-MS	ADNI: 210 NC 178 AD AIBL: 696 NC 268 AD	Strong associations between 218 plasma lipid species and AD	Huynh et al. (2020)

*UPCL* ultra performance liquid chromatography, *HPLC* high performance liquid chromatography, *FUPLC* fast ultrahigh performance liquid chromatography, *QTOF* quadrupole time of flight, *FTICR* fourier transform ion cyclotron resonance spectrometer, *QqQ* triple quadruple, *QTrap* triple quadrupole linear ion trap, *ICP-MS* inductively coupled plasma mass spectrometry, *pCSF* post-mortem cerebrospinal fluid, *ASYMAD* asymptomatic Alzheimer’s disease, *ADNI* Alzheimer’s disease neuroimaging initiative, *BLSA* Baltimore longitudinal study of aging, *FO* framingham offspring, *AIBL* Australian imaging, biomarkers and lifestyle

Another important insight that arose from the reviewed studies, is that alteration of lipids status and related compounds, seem to play a crucial role in AD pathophysiology (Barupal et al., 2019; Casanova et al., 2016; Whiley et al., 2014, Gonzalez-Dominquez et al., 2014; Huynh et al., 2020; Klanvis et al., 2015; Liang et al., 2016; Proitsi et al., 2017; Varma et al., 2018; Wood et al., 2016). Blood lipid profiling using both targeted and untargeted lipidomics-based approaches have been carried out (Astarita et al., 2018; Proitsi et al., 2017; Sandra et al., 2010) and further rigorous studies could actually unlock poor understood biochemical pathway in AD development.

All these data, together with previous available knowledge on the pathophysiological mechanisms involved in AD, could give a huge contribution in the development of reliable diagnostic biomarkers.

In addition, our view is to favor the targeted approaches to validate the previous proposed biomarkers. Many different metabolites have been found to be altered in patients with Alzheimer, however, these potential biomarkers should undergo an external validation process.

Besides, the available metabolomics data should be also integrated with those obtained from clinics, genetics, imaging etc., offering new opportunities to improve diagnosis AD. As recently done by Damotte and co-authors that have built models to accurately distinguish between AD and cognitively normal (CN) individuals (Damotte et al., 2020).

Finally, recently developed imaging mass spectrometry, that combine the screening capability of thousands of metabolites in a single experiment of MS with the possibility of spatial visualization of the selected sample sections is an important analytical tool in clinical investigation (Buchberger et al., 2018; McDonnell & Heeren, 2007). Indeed, MS Imaging has been used for the biological characterization of the lipids changes AD patients in comparison with healthy subjects (Hong et al., 2017; de San Roman et al., 2017). Only with this practical approach we could discard or select appropriate biomarker within those already reported.

## References

[CR1] Alonso A, Marsal S, Julià A (2015). Analytical methods in untargeted metabolomics: State of the art in 2015. Frontiers in Bioengineering and Biotechnology.

[CR2] An Z, Hu T, Lv Y, Li P, Liu L (2020). Targeted amino acid and related amines analysis based on iTRAQ®-LC-MS/MS for discovering potential hepatotoxicity biomarkers. Journal of Pharmaceutical and Biomedical Analysis.

[CR3] Angeloni C, Businaro R, Vauzour D (2020). The role of diet in preventing and reducing cognitive decline. Current Opinion in Psychiatry.

[CR4] Ansoleaga B, Jové M, Schlüter A, Garcia-Esparcia P, Moreno J, Pujol A, Pamplona R, Portero-Otín M, Ferrer I (2015). Deregulation of purine metabolism in Alzheimer's disease. Neurobiology of Aging.

[CR5] Association A (2019). 2019 Alzheimer's disease facts and figures. Alzheimer's & Dementia.

[CR6] Astarita G, Stocchero M, Paglia G (2018). Unbiased lipidomics and metabolomics of human brain samples biomarkers for Alzheimer’s disease drug development.

[CR7] Attems J, Jellinger KA (2014). The overlap between vascular disease and Alzheimer’s disease-lessons from pathology. BMC Medicine.

[CR8] Bailey TL, Rivara CB, Rocher AB, Hof PR (2004). The nature and effects of cortical microvascular pathology in aging and Alzheimer's disease. Neurological Research.

[CR9] Baloni P, Funk CC, Yan J, Yurkovich JT, Kueider-Paisley A, Nho K, Heinken A, Jia W, Mahmoudiandehkordi S, Louie G, Saykin AJ (2020). Metabolic network analysis reveals altered bile acid synthesis and metabolism in Alzheimer’s disease. Cell Reports Medicine.

[CR10] Barupal DK, Baillie R, Fan S, Saykin AJ, Meikle PJ, Arnold M, Nho K, Fiehn O, Kaddurah-Daouk R (2019). Alzheimer's disease neuroimaging initiative and alzheimer disease metabolomics consortium. sets of coregulated serum lipids are associated with alzheimer's disease pathophysiology. Alzheimer's & Dementia.

[CR11] Bracko O, Vinarcsik LK, Hernández JCC, Ruiz-Uribe NE, Haft-Javaherian M, Falkenhain K, Ramanauskaite EM, Ali M, Mohapatra A, Swallow MA, Njiru BN (2020). High fat diet worsens Alzheimer’s disease-related behavioral abnormalities and neuropathology in APP/PS1 mice, but not by synergistically decreasing cerebral blood flow. Scientific Reports.

[CR12] Broadhurst D, Goodacre R, Reinke SN, Kuligowski J, Wilson ID, Lewis MR, Dunn WB (2018). Guidelines and considerations for the use of system suitability and quality control samples in mass spectrometry assays applied in untargeted clinical metabolomic studies. Metabolomics.

[CR13] Buchberger AR, DeLaney K, Johnson J, Li L (2018). Mass spectrometry imaging: A review of emerging advancements and future insights. Analytical Chemistry.

[CR14] Casanova R, Varma S, Simpson B, Kim M, An Y, Saldana S, Riveros C, Moscato P, Griswold M, Sonntag D, Wahrheit J (2016). Blood metabolite markers of preclinical Alzheimer's disease in two longitudinally followed cohorts of older individuals. Alzheimer's & Dementia.

[CR15] Cho Y, Park Y, Sim B, Kim J, Lee H, Cho SN, Kang YA, Lee SG (2020). Identification of serum biomarkers for active pulmonary tuberculosis using a targeted metabolomics approach. Scientific Reports.

[CR16] Chong J, Wishart DS, Xia J (2019). Using metaboanalyst 4.0 for comprehensive and integrative metabolomics data analysis. Current Protocols in Bioinformatics.

[CR17] Chouraki V, Preis SR, Yang Q, Beiser A, Li S, Larson MG, Weinstein G, Wang TJ, Gerszten RE, Vasan RS, Seshadri S (2017). Association of amine biomarkers with incident dementia and Alzheimer's disease in the framingham study. Alzheimer's & Dementia.

[CR18] Corso G, Cristofano A, Sapere N, La Marca G, Angiolillo A, Vitale M, Fratangelo R, Lombardi T, Porcile C, Intrieri M, Di Costanzo A (2017). Serum amino acid profiles in normal subjects and in patients with or at risk of Alzheimer dementia. Dementia and Geriatric Cognitive Disorders Extra.

[CR19] Cristofano A, Sapere N, La Marca G, Angiolillo A, Vitale M, Corbi G, Scapagnini G, Intrieri M, Russo C, Corso G, Di Costanzo A (2016). Serum levels of acyl-carnitines along the continuum from normal to Alzheimer's dementia. PLoS ONE.

[CR20] Czech C, Berndt P, Busch K, Schmitz O, Wiemer J, Most V, Hampel H, Kastler J, Senn H (2012). Metabolite profiling of Alzheimer's disease cerebrospinal fluid. PLoS ONE.

[CR21] Damotte V, Marot G, Meirhaeghe A, Amouyel P, Bellenguez C, Chouraki V, Alzheimer’s Disease Neuroimaging Initiative (2020). Integration of demographics, genetics, imaging and metabolomics data to identify Alzheimer’s disease patients: Neuroimaging/imaging and genetics. Alzheimer's & Dementia.

[CR22] Dawson PH (2013). Quadrupole mass spectrometry and its applications.

[CR98] de San Roman EG, Manuel I, Giralt MT, Ferrer I, Rodríguez-Puertas R (2017). Imaging mass spectrometry (IMS) of cortical lipids from preclinical to severe stages of Alzheimer's disease. Biochimica Et Biophysica Acta (BBA).

[CR23] Di Costanzo A, Paris D, Melck D, Angiolillo A, Corso G, Maniscalco M, Motta A (2020). Blood biomarkers indicate that the preclinical stages of Alzheimer's disease present overlapping molecular features. Scientific Reports.

[CR24] Ding X, Ghobarah H, Zhang X, Jaochico A, Liu X, Deshmukh G, Liederer BM, Hop CE, Dean B (2013). High-throughput liquid chromatography/mass spectrometry method for the quantitation of small molecules using accurate mass technologies in supporting discovery drug screening. Rapid Communications in Mass Spectrometry.

[CR25] Eliuk S, Makarov A (2015). Evolution of orbitrap mass spectrometry instrumentation. Annual Review of Analytical Chemistry.

[CR26] Emwas A-HM, Salek RM, Griffin JL, Merzaban J (2013). NMR-based metabolomics in human disease diagnosis: Applications, limitations, and recommendations. Metabolomics.

[CR27] Farkas E, Luiten PG (2001). Cerebral microvascular pathology in aging and Alzheimer's disease. Progress in Neurobiology.

[CR28] Ferrer I, Martinez A, Boluda S, Parchi P, Barrachina M (2008). Brain banks: Benefits, limitations and cautions concerning the use of post-mortem brain tissue for molecular studies. Cell and Tissue Banking.

[CR29] Fiehn O (2002). Metabolomics—the link between genotypes and phenotypes functional genomics.

[CR30] Forsberg EM, Huan T, Rinehart D, Benton HP, Warth B, Hilmers B, Siuzdak G (2018). Data processing, multi-omic pathway mapping, and metabolite activity analysis using XCMS Online. Nature Protocols.

[CR31] Fortin T, Salvador A, Charrier JP, Lenz C, Bettsworth F, Lacoux X, Choquet-Kastylevsky G, Lemoine J (2009). Multiple reaction monitoring cubed for protein quantification at the low nanogram/milliliter level in nondepleted human serum. Analytical Chemistry.

[CR32] Gelzo M, Clericuzio S, Barone R, D’Apolito O, Russo AD, Corso G (2012). A routine method for cholesterol and 7-dehydrocholesterol analysis in dried blood spot by GC–FID to diagnose the Smith–Lemli–Opitz syndrome. Journal of Chromatography B.

[CR33] Gertsman I, Gangoiti JA, Barshop BA (2014). Validation of a dual LC–HRMS platform for clinical metabolic diagnosis in serum, bridging quantitative analysis and untargeted metabolomics. Metabolomics.

[CR34] González-Domínguez R, García-Barrera T, Gómez-Ariza JL (2014). Combination of metabolomic and phospholipid-profiling approaches for the study of Alzheimer's disease. Journal of Proteomics.

[CR35] Gonzalez-Dominguez R, Javier Ruperez F, García-Barrera T, Barbas C, Luis Gómez-Ariza J (2016). Metabolomic-driven elucidation of serum disturbances associated with Alzheimer’s disease and mild cognitive impairment. Current Alzheimer Research.

[CR36] Goodacre R, Broadhurst D, Smilde AK, Kristal BS, Baker JD, Beger R, Bessant C, Connor S, Capuani G, Craig A, Ebbels T (2007). Proposed minimum reporting standards for data analysis in metabolomics. Metabolomics.

[CR121] Greenberg Nicola, Grassano Antonio, Thambisetty Madhav, Lovestone Simon, Legido-Quigley Cristina (2009). A proposed metabolic strategy for monitoring disease progression in Alzheimer's disease. ELECTROPHORESIS.

[CR37] Gu Y, Nieves JW, Stern Y, Luchsinger JA, Scarmeas N (2010). Food combination and Alzheimer disease risk: A protective diet. Archives of Neurology.

[CR38] Hampel H, O’Bryant SE, Molinuevo JL, Zetterberg H, Masters CL, Lista S, Kiddle SJ, Batrla R, Blennow K (2018). Blood-based biomarkers for Alzheimer disease: Mapping the road to the clinic. Nature Reviews Neurology.

[CR39] Han X, Rozen S, Boyle SH, Hellegers C, Cheng H, Burke JR, Welsh-Bohmer KA, Doraiswamy PM, Kaddurah-Daouk R (2011). Metabolomics in early Alzheimer's disease: Identification of altered plasma sphingolipidome using shotgun lipidomics. PLoS ONE.

[CR40] Han X, Gross RW (2003). Global analyses of cellular lipidomes directly from crude extracts of biological samples by ESI mass spectrometry: A bridge to lipidomics. Journal of Lipid Research.

[CR41] Haraguchi H (2017). Metallomics: The history over the last decade and a future outlook. Metallomics.

[CR42] Hecht ES, Scigelova M, Eliuk S, Makarov A (2006). Fundamentals and advances of orbitrap mass spectrometry. Encyclopedia of Analytical Chemistry.

[CR43] Hong JH, Kang JW, Kim DK, Baik SH, Kim KH, Shanta SR, Jung JH, Mook-Jung I, Kim KP (2016). Global changes of phospholipids identified by MALDI imaging mass spectrometry in a mouse model of Alzheimer's disease. Journal of Lipid Research.

[CR44] Huan T, Forsberg EM, Rinehart D, Johnson CH, Ivanisevic J, Benton HP, Fang M, Aisporna A, Hilmers B, Poole FL, Thorgersen MP (2017). Systems biology guided by XCMS online metabolomics. Nature Methods.

[CR45] Huan T, Tran T, Zheng J, Sapkota S, MacDonald SW, Camicioli R, Dixon RA, Li L (2018). Metabolomics analyses of saliva detect novel biomarkers of Alzheimer’s disease. Journal of Alzheimer's Disease.

[CR46] Huang S, Guo Y, Li Z, Zhang Y, Zhou T, You W, Pan K, Li W (2020). A systematic review of metabolomic profiling of gastric cancer and esophageal cancer. Cancer Biology & Medicine.

[CR47] Huang Y, Mucke L (2012). Alzheimer mechanisms and therapeutic strategies. Cell.

[CR48] Huynh K, Lim WLF, Giles C, Jayawardana KS, Salim A, Mellett NA, Smith AAT, Olshansky G, Drew BG, Chatterjee P, Martins I (2020). Concordant peripheral lipidome signatures in two large clinical studies of Alzheimer’s disease. Nature Communications.

[CR49] Iannuzzi F, Sirabella R, Canu N, Maier TJ, Annunziato L, Matrone C (2020). Fyn tyrosine kinase elicits amyloid precursor protein Tyr682 phosphorylation in neurons from Alzheimer’s disease patients. Cells.

[CR50] Ibanez C, Simo C, Martin-Alvarez PJ, Kivipelto M, Winblad B, Cedazo-Minguez A, Cifuentes A (2012). Toward a predictive model of Alzheimer's disease progression using capillary electrophoresis-mass spectrometry metabolomics. Analytical Chemistry.

[CR51] Inoue K, Tsutsui H, Akatsu H, Hashizume Y, Matsukawa N, Yamamoto T, Toyo'Oka T (2013). Metabolic profiling of Alzheimer's disease brains. Scientific Reports.

[CR52] Irvine GW, Nguyen S (2019). An overview of the “-omics” fields at the forefront of next-generation personalized medicine and fundamental systems biology studies. Biomedical Genetics and Genomics.

[CR53] Jack CR, Bennett DA, Blennow K, Carrillo MC, Dunn B, Haeberlein SB, Holtzman DM, Jagust W, Jessen F, Karlawish J, Liu E (2018). NIA-AA research framework: Toward a biological definition of Alzheimer's disease. Alzheimer's & Dementia.

[CR54] John-Williams St L, Blach C, Toledo JB, Rotroff DM, Kim S, Klavins K, Baillie R, Han X, Mahmoudiandehkordi S, Jack J, Massaro TJ (2017). Targeted metabolomics and medication classification data from participants in the ADNI1 cohort. Scientific Data.

[CR55] Klassen A, Faccio AT, Canuto GAB, da Cruz PLR, Ribeiro HC, Tavares MFM, Sussulini A (2017). Metabolomics: Definitions and significance in systems biology. Metabolomics.

[CR56] Klavins K, Koal T, Dallmann G, Marksteiner J, Kemmler G, Humpel C (2015). The ratio of phosphatidylcholines to lysophosphatidylcholines in plasma differentiates healthy controls from patients with Alzheimer's disease and mild cognitive impairment. Alzheimer's & Dementia.

[CR57] Kohler I, Verhoeven A, Derks RJ, Giera M (2016). Analytical pitfalls and challenges in clinical metabolomics. Bioanalysis.

[CR58] La Rosa LR, Perrone L, Nielsen MS, Calissano P, Andersen OM, Matrone C (2015). Y682G mutation of amyloid precursor protein promotes endo-lysosomal dysfunction by disrupting APP–SorLA interaction. Frontiers in Cellular Neuroscience.

[CR59] Lapthorn C, Pullen F, Chowdhry BZ (2013). Ion mobility spectrometry-mass spectrometry (IMS-MS) of small molecules: Separating and assigning structures to ions. Mass Spectrometry Reviews.

[CR60] Leonenko G, Shoai M, Bellou E, Sims R, Williams J, Hardy J, Escott-Price V, Initiative ADN (2019). Genetic risk for alzheimer disease is distinct from genetic risk for amyloid deposition. Annals of Neurology.

[CR61] Li NJ, Liu WT, Li W, Li SQ, Chen XH, Bi KS, He P (2010). Plasma metabolic profiling of Alzheimer's disease by liquid chromatography/mass spectrometry. Clinical Biochemistry.

[CR62] Li S (2020). Computational methods and data analysis for metabolomics.

[CR63] Li S, Mou H, Jiang N (2019). Application of high performance liquid chromatography-quadruple/linear ion trap mass spectrometry in food analysis. Journal of Food Safety and Quality.

[CR64] Liang Q, Liu H, Li X, Zhang AH (2016). High-throughput metabolomics analysis discovers salivary biomarkers for predicting mild cognitive impairment and Alzheimer's disease. RSC Advances.

[CR65] Lindon JC, Nicholson JK, Everett JR (1999). NMR spectroscopy of biofluids annual reports on NMR spectroscopy.

[CR66] Liu P, Fleete MS, Jing Y, Collie ND, Curtis MA, Waldvogel HJ, Faull RL, Abraham WC, Zhang H (2014). Altered arginine metabolism in Alzheimer's disease brains. Neurobiology of Aging.

[CR67] Liu W, Song Q, Cao Y, Zhao Y, Huo H, Wang Y, Song Y, Li J, Tu P (2019). Advanced liquid chromatography-mass spectrometry enables merging widely targeted metabolomics and proteomics. Analytica Chimica Acta.

[CR68] Long NP, Nghi TD, Kang YP, Anh NH, Kim HM, Park SK, Kwon SW (2020). Toward a standardized strategy of clinical metabolomics for the advancement of precision medicine. Metabolites.

[CR69] Love S, Miners JS (2016). Cerebrovascular disease in ageing and Alzheimer’s disease. Acta Neuropathologica.

[CR70] Lu W, Bennett BD, Rabinowitz JD (2008). Analytical strategies for LC–MS-based targeted metabolomics. Journal of Chromatography B.

[CR71] Mahmoudian Dehkordi S, Arnold M, Nho K, Ahmad S, Jia W, Xie G, Louie G, Kueider-Paisley A, Moseley MA, Thompson JW, Williams LSJ (2019). Altered bile acid profile associates with cognitive impairment in Alzheimer's disease—An emerging role for gut microbiome. Alzheimer's & Dementia.

[CR72] Mapstone M, Cheema AK, Fiandaca MS, Zhong X, Mhyre TR, MacArthur LH, Hall WJ, Fisher SG, Peterson DR, Haley JM, Nazar MD (2014). Plasma phospholipids identify antecedent memory impairment in older adults. Nature Medicine.

[CR73] Marksteiner J, Blasko I, Kemmler G, Koal T, Humpel C (2018). Bile acid quantification of 20 plasma metabolites identifies lithocholic acid as a putative biomarker in Alzheimer’s disease. Metabolomics.

[CR74] Marshall AG, Hendrickson CL (2008). High-resolution mass spectrometers. Annual Review in Analitical Chemistry.

[CR75] Matrone C (2013). A new molecular explanation for age-related neurodegeneration: The Tyr682 residue of amyloid precursor protein. BioEssays.

[CR76] Matrone C, Annunziato L, Iannuzzi F (2019). The Y682ENPTY687 motif of app: Progress and insights toward a targeted therapy for Alzheimer’s disease patients. Ageing Research Reviews.

[CR77] Matrone C, Ciotti MT, Mercanti D, Marolda R, Calissano P (2008). NGF and BDNF signaling control amyloidogenic route and Aβ production in hippocampal neurons. Proceedings of the National Academy of Sciences.

[CR78] Matrone C, Marolda R, Ciafrè S, Ciotti M, Mercanti D, Calissano P (2009). Tyrosine kinase nerve growth factor receptor switches from prosurvival to proapoptotic activity via Abeta-mediated phosphorylation. Proceedings of the National Academy of Sciences.

[CR79] Matrone C, Petrillo F, Nasso R, Ferretti G (2020). Fyn tyrosine kinase as harmonizing factor in neuronal functions and dysfunctions. International Journal of Molecular Sciences.

[CR80] McDonnell LA, Heeren RM (2007). Imaging mass spectrometry. Mass Spectrometry Reviews.

[CR122] Mesa Sanchez D, Creger S, Singla V, Kurulugama RT, Fjeldsted J, Laskin Julia (2020). Ion Mobility-Mass Spectrometry Imaging Workflow. Journal of the American Society for Mass Spectrometry.

[CR81] Muguruma Y, Tsutsui H, Noda T, Akatsu H, Inoue K (2018). Widely targeted metabolomics of Alzheimer's disease postmortem cerebrospinal fluid based on 9-fluorenylmethyl chloroformate derivatized ultra-high performance liquid chromatography tandem mass spectrometry. Journal of Chromatography B.

[CR82] Navas-Carrillo D, Rivera-Caravaca JM, Sampedro-Andrada A, Orenes-Piñero E (2020). Novel biomarkers in Alzheimer’s disease using high resolution proteomics and metabolomics: miRNAS, proteins and metabolites. Critical Reviews in Clinical Laboratory Sciences.

[CR83] Nho K, Kueider-Paisley A, MahmoudianDehkordi S, Arnold M, Risacher SL, Louie G, Blach C, Baillie R, Han X, Kastenmüller G, Jia W (2019). Altered bile acid profile in mild cognitive impairment and Alzheimer's disease: Relationship to neuroimaging and CSF biomarkers. Alzheimer's & Dementia.

[CR84] Oakley H, Cole SL, Logan S, Maus E, Shao P, Craft J, Guillozet-Bongaarts A, Ohno M, Disterhoft J, Van Eldik L, Berry R (2006). Intraneuronal β-amyloid aggregates, neurodegeneration, and neuron loss in transgenic mice with five familial Alzheimer's disease mutations: Potential factors in amyloid plaque formation. Journal of Neuroscience.

[CR85] Oresic M, Hyotylainen T, Herukka SK, Sysi-Aho M, Mattila I, Seppanan-Laakso T, Julkunen V, Gopalacharyulu PV, Hallikainen M, Koikkalainen J, Kivipelto M, Helisalmi S, Lotjonen J, Soininen H (2011). Metabolome in progression to Alzheimer's disease. Translational Psychiatry.

[CR86] Pagani M, Nobili F, Morbelli S, Arnaldi D, Giuliani A, Öberg J, Girtler N, Brugnolo A, Picco A, Bauckneht M, Piva R (2017). Early identification of MCI converting to AD: A FDG PET study. European Journal of Nuclear Medicine and Molecular Imaging.

[CR87] Paglia G, Kliman M, Claude E, Geromanos S, Astarita G (2015). Applications of ion-mobility mass spectrometry for lipid analysis. Analytical and Bioanalytical Chemistry.

[CR88] Paglia G, Miedico O, Cristofano A, Vitale M, Angiolillo A, Chiaravalle AE, Corso G, Di Costanzo A (2016). Distinctive pattern of serum elements during the progression of Alzheimer’s disease. Scientific Reports.

[CR89] Paglia G, Stocchero M, Cacciatore S, Lai S, Angel P, Alam MT, Keller M, Ralser M, Astarita G (2016). Unbiased metabolomic investigation of Alzheimer’s disease brain points to dysregulation of mitochondrial aspartate metabolism. Journal of Proteome Research.

[CR90] Pinto FG, Mahmud I, Harmon TA, Rubio VY, Garrett TJ (2020). Rapid prostate cancer noninvasive biomarker screening using segmented flow mass spectrometry-based untargeted metabolomics. Journal of Proteome Research.

[CR91] Poulsen ET, Iannuzzi F, Rasmussen HF, Maier TJ, Enghild JJ, Jørgensen AL, Matrone C (2017). An aberrant phosphorylation of amyloid precursor protein tyrosine regulates its trafficking and the binding to the clathrin endocytic complex in neural stem cells of Alzheimer's Disease Patients. Frontiers in Molecular Neuroscience.

[CR92] Poulsen E, Larsen A, Zollo A, Jørgensen AL, Sanggaard KW, Enghild JJ, Matrone C (2015). New insights to clathrin and adaptor protein 2 for the design and development of therapeutic strategies. International Journal of Molecular Sciences.

[CR93] Proitsi P, Kim M, Whiley L, Simmons A, Sattlecker M, Velayudhan L, Lupton MK, Soininen H, Kloszewska I, Mecocci P, Tsolaki M (2017). Association of blood lipids with Alzheimer's disease: A comprehensive lipidomics analysis. Alzheimer's & Dementia.

[CR94] Purandare N, Zubair M, Xu Y, Broadhurst D, Dunn WB, Begley P, Francis-McIntyre S, Chew-Graham S, Halsall A, Consortium H, Burns A (2009). P4–310: Serum metabolite biomarkers in Alzheimer's disease. Alzheimer's & Dementia.

[CR95] Rahman A, Schelbaum E, Hoffman K, Diaz I, Hristov H, Andrews R, Jett S, Jackson H, Lee A, Sarva H, Pahlajani S (2020). Sex-driven modifiers of Alzheimer risk: A multimodality brain imaging study. Neurology.

[CR96] Rami L, Bosch B, Sanchez-Valle R, Molinuevo J (2010). The memory alteration test (M@ T) discriminates between subjective memory complaints, mild cognitive impairment and Alzheimer's disease. Archives of Gerontology and Geriatrics.

[CR97] Reiman EM (2006). Focus on Alzheimer's disease and related disorders: A 100-year update on Alzheimer's disease and related disorders. The Journal of Clinical Psychiatry.

[CR99] Sandra K, dos Santos Pereira A, Vanhoenacker G, David F, Sandra P (2010). Comprehensive blood plasma lipidomics by liquid chromatography/quadrupole time-of-flight mass spectrometry. Journal of Chromatography A.

[CR100] Shi W, Chance MR (2008). Metallomics and metalloproteomics. Cellular and Molecular Life Sciences.

[CR101] Shishtar E, Rogers GT, Blumberg JB, Au R, Jacques PF (2020). Long-term dietary flavonoid intake and risk of Alzheimer disease and related dementias in the framingham offspring cohort. The American Journal of Clinical Nutrition.

[CR102] Sperling R, Mormino E, Johnson K (2014). The evolution of preclinical Alzheimer’s disease: Implications for prevention trials. Neuron.

[CR103] Sumner LW, Amberg A, Barrett D, Beale MH, Beger R, Daykin CA, Fan TWM, Fiehn O, Goodacre R, Griffin JL, Hankemeier T (2007). Proposed minimum reporting standards for chemical analysis. Metabolomics.

[CR104] Sussulini A (2017). Metabolomics: from fundamentals to clinical applications.

[CR105] Takayama T, Mizuno H, Toyo’oka T, Akatsu H, Inoue K, Todoroki K (2019). Isotope corrected chiral and achiral nontargeted metabolomics: An approach for high accuracy and precision metabolomics based on derivatization and its application to cerebrospinal fluid of patients with Alzheimer’s disease. Analytical Chemistry.

[CR106] Takayama T, Mochizuki T, Todoroki K, Min JZ, Mizuno H, Inoue K, Akatsu H, Noge I, Toyo'oka T (2015). A novel approach for LC-MS/MS-based chiral metabolomics fingerprinting and chiral metabolomics extraction using a pair of enantiomers of chiral derivatization reagents. Analytica Chimica Acta.

[CR107] Tang Y, Zhu Y, Sang S (2020). A novel LC-MS based targeted metabolomic approach to study the biomarkers of food intake. Molecular Nutrition & Food Research.

[CR108] Trushina E, Dutta T, Persson X-MT, Mielke MM, Petersen RC (2013). Identification of altered metabolic pathways in plasma and CSF in mild cognitive impairment and Alzheimer’s disease using metabolomics. PLoS ONE.

[CR109] Van Cauwenberghe C, Van Broeckhoven C, Sleegers K (2016). The genetic landscape of Alzheimer disease: Clinical implications and perspectives. Genetics in Medicine.

[CR110] Varma VR, Oommen AM, Varma S, Casanova R, An Y, Andrews RM, O’Brien R, Pletnikova O, Troncoso JC, Toledo J, Baillie R (2018). Brain and blood metabolite signatures of pathology and progression in Alzheimer disease: A targeted metabolomics study. PLoS Medicine.

[CR111] Veurink G, Perry G, Singh SK (2020). Role of antioxidants and a nutrient rich diet in Alzheimer's disease. Open Biology.

[CR112] Wang G, Zhou Y, Huang FJ, Tang HD, Xu XH, Liu JJ, Wang Y, Deng YL, Ren RJ, Xu W, Ma JF (2014). Plasma metabolite profiles of Alzheimer’s disease and mild cognitive impairment. Journal of Proteome Research.

[CR113] Wang J, Wang C, Han X (2019). Tutorial on lipidomics. Analytica Chimica Acta.

[CR114] Whiley L, Sen A, Heaton J, Proitsi P, García-Gómez D, Leung R, Smith N, Thambisetty M, Kloszewska I, Mecocci P, Soininen H (2014). Evidence of altered phosphatidylcholine metabolism in Alzheimer's disease. Neurobiology of Aging.

[CR115] Wilkins JM, Trushina E (2018). Application of metabolomics in Alzheimer’s disease. Frontiers in Neurology.

[CR116] Wood PL, Locke VA, Herling P, Passaro A, Vigna GB, Volpato S, Valacchi G, Cervellati C, Zuliani G (2016). Targeted lipidomics distinguishes patient subgroups in mild cognitive impairment (MCI) and late onset Alzheimer's disease (LOAD). BBA Clinical.

[CR117] Xia J, Broadhurst DI, Wilson M, Wishart DS (2013). Translational biomarker discovery in clinical metabolomics: An introductory tutorial. Metabolomics.

[CR118] Yin P, Xu G (2014). Current state-of-the-art of nontargeted metabolomics based on liquid chromatography–mass spectrometry with special emphasis in clinical applications. Journal of Chromatography A.

[CR119] Zhang S, Wang Z, Cai F, Zhang M, Wu Y, Zhang J, Song W (2017). BACE1 cleavage site selection critical for amyloidogenesis and Alzheimer's pathogenesis. Journal of Neuroscience.

[CR120] Zhou J, Yin Y (2016). Strategies for large-scale targeted metabolomics quantification by liquid chromatography-mass spectrometry. The Analyst.

